# Acute kidney injury is associated with abnormal cefepime exposure among critically ill children and young adults

**DOI:** 10.1007/s00467-024-06477-4

**Published:** 2024-08-16

**Authors:** Kathryn Pavia, Sonya Tang Girdwood, Kelli Paice, Min Dong, Tomoyuki Mizuno, Peter Tang, Colleen Mangeot, Alexander A. Vinks, Jennifer Kaplan

**Affiliations:** 1 Division of Critical Care Medicine, Cincinnati Children’s Hospital Medical Center, Cincinnati, OH USA; 2 Division of Translational and Clinical Pharmacology, Cincinnati Children’s Hospital Medical Center, Cincinnati, OH 45229 USA; 3 Division of Hospital Medicine, Cincinnati Children’s Hospital Medical Center, Cincinnati, OH USA; 4 Department of Pediatrics, University of Cincinnati College of Medicine, Cincinnati, OH USA; 5 Division of Pathology and Laboratory Medicine, Cincinnati Children’s Hospital Medical Center, Cincinnati, OH USA; 6 Division of Biostatistics and Epidemiology, Cincinnati Children’s Hospital Medical Center, Cincinnati, OH USA

**Keywords:** Cefepime, Acute kidney injury, Pharmacokinetics precision dosing

## Abstract

**Background:**

Elevated cefepime blood concentrations can cause neurotoxicity in adults. The consequences of elevated cefepime concentrations among pediatric patients are unknown. Future exploration of such effects requires first identifying patients at risk for elevated cefepime exposure. We investigated the role of acute kidney injury as a risk factor for increased cefepime concentrations in critically ill children.

**Methods:**

This was a retrospective analysis at a single pediatric intensive care unit. Analyzed patients received at least 24 h of cefepime and had at least two opportunistic samples collected for total cefepime concentration measurement. Individual pharmacokinetic (PK) profiles during treatment courses were reconstructed using Bayesian estimation with an established population PK model. Elevated trough concentration (*C*_min_) was defined as ≥ 30 mg/L based on adult toxicity studies. The effect of kidney dysfunction on cefepime PK profiles was interrogated using a mixed-effect model.

**Results:**

Eighty-seven patients were included, of which 13 (14.9%) had at least one estimated *C*_min_ ≥ 30 mg/L. Patients with elevated *C*_min_ were more likely to have acute kidney injury (AKI) during their critical illness (92% vs. 57%, *p* = 0.015 for any AKI; 62% vs. 26%, *p* = 0.019 for severe AKI). Patients who had AKI during critical illness had significantly higher cefepime exposure, as quantified by the area under the concentration–time curve over 24 h (AUC_24h_) and *C*_min_.

**Conclusions:**

Among critically ill children, AKI is associated with elevated cefepime concentrations. Identifying these high-risk patients is the first step toward evaluating the clinical consequences of such exposures.

**Graphical abstract:**

**Figure:**
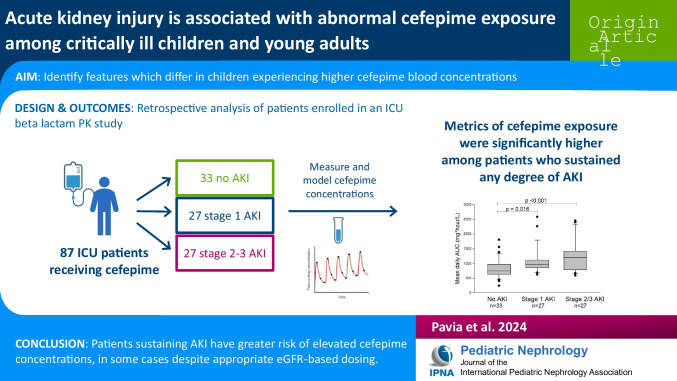
A higher resolution version of the Graphical abstract is available as Supplementary information

**Supplementary Information:**

The online version contains supplementary material available at 10.1007/s00467-024-06477-4.

## Background

Sepsis and septic shock are common reasons for PICU admission and carry a substantial mortality burden [1]. Timely antibiotic administration is a cornerstone of sepsis management. However, physiologic derangements in critical illness make antibiotic dosing in this context more challenging. Fluid overload and capillary leak may increase the volume of distribution, and augmented kidney clearance may lead to more rapid elimination [2, 3]. Conversely, dysfunction or failure of organs like the liver or kidneys may result in decreased medication clearance, requiring a reduction in antibiotic dose or dosing frequency to avoid toxicity. Patients with sepsis are at increased risk of developing AKI [4]. Between 10 and 25% of pediatric patients with sepsis develop AKI, and the incidence increases with increased sepsis severity [5, 6]. This high incidence of AKI in sepsis must be considered when dosing medications which are eliminated via the kidneys.

Cefepime is a fourth-generation beta-lactamase–resistant cephalosporin with a broad range of activity against gram-positive and gram-negative organisms, including *Pseudomonas aeruginosa* [7]*.* It is among the most commonly prescribed antibiotics for hospital-acquired infections [8]. Cefepime has approximately 15–20% protein binding [9], a volume of distribution of 15–30 L in adults [10], and clearance primarily via excretion into the urine in its unchanged form [11]. Cefepime has not been shown to have a direct impact on kidney function, but due to these elimination properties, it is susceptible to substantial changes in pharmacokinetics (PK) during periods of altered kidney physiology such as critical illness [12], and requires dose adjustments based on kidney function and use of kidney replacement therapies [13]. There is a paucity of PK data to inform cefepime dosing in pediatric patients in the setting of varying kidney function [14, 15], such that recommended dose adjustments are frequently based on expert opinion rather than prospective clinical trials. Changes in body composition and organ maturation throughout the neonatal and pediatric periods further complicate attempts to design unified dosing recommendations for infants and children [16]. Measurement of cefepime concentrations is not routinely available outside of the research context in most of the United States, although implementation of therapeutic drug monitoring has been described [17, 18].

Cefepime has excellent central nervous system penetration which is clinically useful for treating intracranial infections [19], but also facilitates cefepime’s neurologic side effects. Elevated plasma cefepime concentrations in adults have been associated with neurological symptoms ranging from confusion to seizures [20]. Patients experiencing cefepime-induced neurotoxicity consistently have elevated cefepime concentrations compared to those who do not experience neurotoxic effects [20], although there is no consensus concentration threshold for such toxicity. Assorted studies in adults have proposed thresholds for increased neurologic events at trough concentrations ranging from 20 to 50 mg/L [21–23]. Investigation of cefepime concentrations associated with neurotoxicity among children has been limited to case reports [24, 25].

At our institution, critically ill children’s cefepime trough concentrations vary up to 16-fold (range 5–86 mg/L) with standard dosing regimens [26]. This large between-patient variability places some patients at risk of under-exposure and ineffective antibacterial activity and others at risk of over-exposure and thus toxic side effects. Here, we sought to characterize demographic and illness factors associated with elevated cefepime trough concentrations among critically ill children and to interrogate the impact of kidney dysfunction as a risk factor for impaired cefepime clearance.

## Methods

### *Patient selection and enrollment*

Patients at a single center were selected from those enrolled as part of an ongoing study of pharmacokinetics and pharmacodynamics of beta-lactam antibiotics in PICU patients. The study was approved on 9/2/2018 by the Cincinnati Children’s Institutional Review Board under study number 2018–3245 as “BetaLactamPKSepsis.” It was granted a waiver of consent and was conducted in compliance with the ethical standards of the responsible committee on human experimentation and with the Helsinki Declaration of 1975. Briefly, patients who received at least one dose of cefepime in the PICU during the study collection periods were sequentially screened for inclusion. Patients were included if they met the following criteria: (1) at least one dose of cefepime administered in the PICU with a full 24 h of pharmacokinetic data, (2) PICU stay of duration ≥ 24 h, and (3) at least two opportunistic plasma samples available and processed within 72 h of collection. Once enrolled, patients could not be re-enrolled during the same hospitalization but were eligible for repeat enrollment with subsequent admissions. Prior or concurrent administration of other antibiotics did not exclude patients from enrollment. Patients who were on extracorporeal support such as extracorporeal membrane oxygenation (ECMO) or continuous kidney replacement therapy (CKRT) at the time of sample collection were excluded from this analysis. To focus on the impact of acute kidney injury, patients with baseline eGFR < 60 mL/min/1.73 m^2^ (i.e., CKD stages 3–5) as identified by chart review of the 6 months prior to study enrollment were excluded in the primary analysis. Demographics of the population with five CKD patients included may be found in Supplemental Table S1.

### Sample collection and processing

Blood samples were obtained using scavenged residual samples with an opportunistic sampling strategy, as described previously [26, 27]. After completion of clinically ordered testing, residual blood collected in ethylenediaminetetraacetic acid (EDTA) or lithium-heparin tubes was requisitioned from the clinical laboratory. Samples collected within 30 min of initiation of cefepime administration were excluded, as a 30-min infusion is standard at our institution. Samples were requested daily until cefepime discontinuation, hospital discharge, or death, up to a maximum of 7 days. For the second 50 patients (enrolled 9/2021–12/2021), sample collection was discontinued upon transfer out of the ICU, up to a maximum of 7 days. Samples were stored at 4 °C for up to 72 h from the time of collection until processing and then centrifuged for 10 min at 2060 × g, after which supernatant was isolated and frozen at − 80 °C until cefepime concentration measurement. Total cefepime concentrations were measured using linear gradient high-performance liquid chromatography as described previously [26].

### Chart review

Electronic medical records (EMR) were reviewed, and clinical data were collected including patient demographics, medical conditions, features of hospitalization, and identified infections. Body mass index (BMI) categories were assessed based on 2000 Center for Disease Control weight-for-length growth charts for children 0–3 years, BMI growth charts for patients 3–20 years, and a Center for Disease Control BMI calculator for patients over 20 years. Following CDC categorizations, underweight was defined as patients < 5th percentile for age, while overweight/obese was defined as > 85th percentile for age. All creatinine measurements were obtained via chart review. Creatinine measurements used for clinical care are performed by our CLIA- and CAP-certified Cincinnati Children’s Clinical Laboratory via a validated biochromatic assay. Baseline serum creatinine was defined as the lowest recorded creatinine within 3 months prior to hospitalization. For patients with prolonged hospitalization, the lowest creatinine within current hospitalization prior to ICU admission was used. For patients without prior creatinine data in our system, the baseline was the lowest value of either lowest creatinine during current hospitalization or imputed creatinine for a glomerular filtration rate (GFR) of 120 mL/min/1.73 m^2^ using the modified bedside Schwartz equation with *k* = 0.413 [28, 29]. Estimated GFR was calculated using Chronic Kidney Disease in Children (CKiD U25) calculating equations via the published shiny app [30]. Daily patient data were reviewed starting from the first day on which cefepime was administered in the ICU and continuing until cessation of cefepime sample monitoring.

### Modeling

Bayesian PK analysis was performed with MwPharm +  + (Mediware, Prague, Czech Republic). Using a previously published two-compartment model with allometric scaling describing cefepime population PK in critically ill pediatric patients [14] along with the measured cefepime concentrations, Bayesian estimation was used to generate a concentration–time profile for each patient. Estimates for the clearance, volume of distribution, area under the concentration–time curve (AUC), and minimum/maximum concentrations in each dosing interval (*C*_min_/*C*_max_) were extracted. The volume of distribution was linearly scaled, and clearance was allometrically scaled with a power of 0.75 to patient body weight, as described previously [14].

### Acute kidney injury analysis

AKI stage for each day of study observation was staged using Kidney Disease Defining Global Outcomes (KDIGO) creatinine criteria only [31]. The highest serum creatinine measured within each 24-h period was chosen.

### Dosing and dose adjustments

The electronic health record was used to collect the cefepime doses ordered for all patients with severe (KDIGO stages 2–3) AKI. Cefepime dosing and adjustments were managed by the clinical team and were not influenced by study inclusion. Institutional dosing recommendations are included in Supplemental Table S3 [32]. Intra-operative dosing was not reported due to different medication reporting contexts within the EMR.

### Statistics and analysis

A pre-dose trough concentration (*C*_min_) of 30 mg/L was chosen as the threshold for “elevated” cefepime exposure based on a previous adult analysis [22]. Continuous data were compared using Mann–Whitney rank sum tests, and categorical data were compared using chi-squared analysis. For analysis of the AKI effect on cefepime PK parameters and exposure, groups were analyzed by one-way ANOVA for continuous data and chi-squared analysis for categorical data, with pairwise comparisons using Dunn’s methods. For these analyses, patients were categorized by the highest degree of AKI achieved at any point during the study observation period, with the categories being no AKI, stage 1 AKI, and severe AKI (stages 2 and 3). To account for potential confounders, a mixed-effect model was used to investigate the effect of the AKI stage on cefepime exposure measured both by *C*_min_ and AUC_24_. Three patient-level variables were added to the mixed model: age in years, Pediatric RISk of Mortality (PRISM) score, and baseline eGFR. As the data were not normally distributed, analysis was performed with both original and log-transformed data. The outcomes of the analysis were similar for both, so non-transformed results are presented for ease of interpretation. Statistical calculations were performed using SigmaPlot V.15 (Inpixion) and SPSS (IBM).

## Results

A total of 87 patients were included with a total of 488 samples available for analysis (2–20 samples per patient). The demographics of the cohort are described in Table 1. Patients with chronic kidney disease were excluded for the primary analysis of this study, but the complete population is included in Supplemental Table S1. All ages were included in this analysis, but a sub-analysis of patients < 18 years of age (*n* = 70) is included for comparison in Supplemental Table S2. The baseline eGFR of 167 mL/min/1.73m^2^ is higher than that expected for a healthy pediatric population but similar to that reported in other international studies of critically ill patients [33]. The median number of concurrent medications per patient was 11 and vancomycin was co-administered on 36% of all patient-days.

**Table 1:** Patient demographics and features of hospitalization. Results are reported as median (interquartile range) for non-normally distributed continuous variables, and *n* (%) for categorical variables. Groups were analyzed by the Mann–Whitney test for continuous and chi-squared or Fisher’s exact analysis for categorical variables. Weight was recorded at PICU admission. BMI was calculated using CDC BMI percentile growth charts. Baseline creatinine represents the lowest within 3 months prior to hospitalization. Ventilator days represent invasive or non-invasive positive pressure ventilation. For patients with baseline positive pressure ventilation, the duration of support level greater than their baseline is reported. Vasopressor days include any day on which the patient received continuous infusions of epinephrine, norepinephrine, dopamine, vasopressin, or milrinone. *eGFR*, estimated glomerular filtration rate; *LOS*, length of stay; *PICU*, pediatric intensive care unit; *PRISM*, Pediatric RISk of Mortality

	All patients (*N* = 87)	At least 1, *C*_min_ ≥ 30 mg/L (*n* = 13)	All, *C*_min_ < 30 mg/L (*n* = 74)	Comparison (*p* value)
Demographics				
Age, years	8.3 (2–17.7)	10.4 (3.5–21.1)	7.5 (2.0–16.0)	0.28
Age in years, *n* (%)				0.03
0– < 2	21 (29%)	3 (23%)	18 (25%)	
2– < 6	17 (23%)	1 (8%)	16 (22%)	
6– < 12	13 (18%)	3 (23%)	10 (14%)	
12– < 18	19 (26%)	0 (0%)	19 (26%)	
≥ 18	17 (23%)	6 (46%)	11 (15%)	
Weight, kg	24.9 (12.7–54.7)	32.7 (13.8–51.9)	23.8 (12.6–56.0)	0.87
BMI category				
Underweight, *n* (%)	10 (11%)	2 (15%)	8 (11%)	0.71
Healthy weight, *n* (%)	49 (57%)	8 (62%)	41 (46%)	
Overweight or obese, *n* (%)	28 (33%)	3 (23%)	25 (34%)	
Sex, identified at birth *n*, male (%)	49 (56%)	7 (54%)	42 (57%)	0.91
Race/ethnicity				
White, *n* (%)	62 (71%)	12 (92%)	50 (68%)	0.09
Non-white or unknown, *n* (%)	25 (29%)	1 (8%)	24 (32%)	
Baseline creatinine (mg/dL)	0.26 (0.17–0.42)	0.33 (0.17–0.49)	0.26 (0.17–0.39)	0.34
Baseline eGFR by CKiD U25 (mL/min/1.73m^2^)	167 (134–231)	130 (119–226)	171 (142–232)	0.13
Oncologic diagnosis	23 (26%)	3 (23%)	20 (27%)	1.00
Solid organ or bone marrow transplant	17 (20%)	5 (38%)	12 (16%)	0.12
Characteristics of hospitalization				
Hospital LOS, days	28 (11–68)_	20 (11.5–107.5)	30 (11–66)	0.78
PICU LOS, days	8 (4–17)	9 (4.5–20)	7.5 (3–17)	0.50
Ventilator days	3 (0–12)	4 (0–16.5)	2 (0–11.25)	0.49
Vasopressor days	0 (0–1)	1 (0–1)	0 (0–1)	0.29
Any AKI	54 (62%)	12 (92%)	42 (57%)	0.015
Stages 2–3 AKI	27 (31%)	8 (62%)	19 (26%)	0.019
PRISM score	5 (2–11)	6 (2–17)	4 (2–10)	0.44
Total duration cefepime therapy, days	6 (308)	7 (4–10.5)	5 (3–8)	0.25
28-day mortality	5 (6%)	2 (15%)	3 (4%)	0.15

### Predictors of elevated cefepime exposure

A threshold of 30 mg/L was chosen for initial analyses of “elevated” cefepime concentrations based on an adult study predicting a 95% likelihood of neurological side effects when plasma trough concentrations exceed 30 mg/L [22]. Thirteen patients (14.9%) had at least one *C*_min_ greater than 30 mg/L, while the remaining 74 (86.1%) did not (Table 1). Patients who developed elevated *C*_min_ typically did so early in the study: 8 (62%) had first recorded *C*_min_ ≥ 30 within the first 24 h of study enrollment, 4 (31%) had the first *C*_min_ ≥ 30 between 24 and 48 h, and only 1 (8%) had a first *C*_min_ ≥ 30 at ≥ 72 h (Supplemental Figure S1). Patient and clinical factors were compared to identify predictors of patients who developed elevated concentrations. Patients with elevated *C*_min_ had similar demographics and non-kidney-related comorbid diagnoses compared to those without. The median age did not differ between groups, although the distribution of age categories was different (*p* = 0.03). There was no statistical difference in baseline eGFR prior to critical illness. During their hospitalization, patients with elevated cefepime *C*_min_ concentrations had similar markers of illness severity as those without elevated *C*_min_. Patients with elevated cefepime *C*_min_ concentrations had higher rates of AKI (92% vs. 57%, *p* = 0.015).

### Impact of AKI on cefepime exposure

**Fig. 1:**
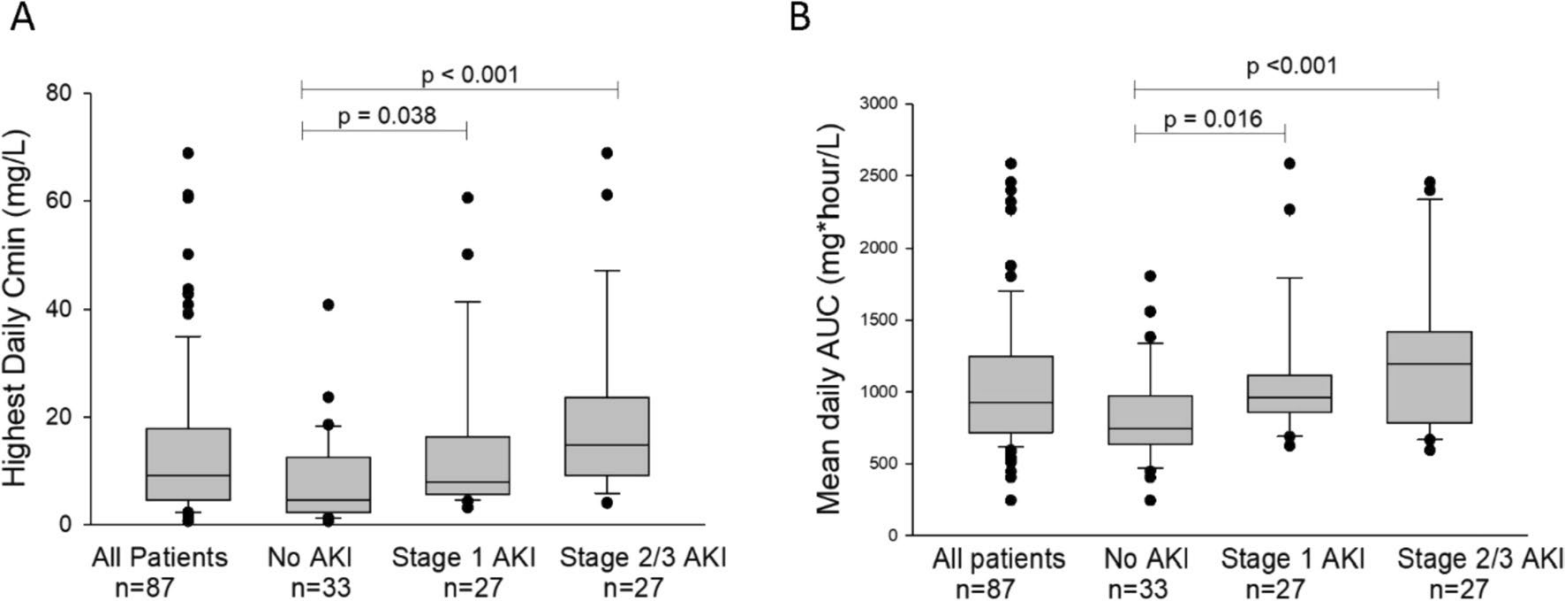
Pairwise comparisons of cefepime exposure based on maximum AKI stage. Pairwise comparisons between different AKI categories performed using Dunn’s method. Comparison of **A** highest daily *C*_min_ and **B** AUC_24_

Due to both biological plausibility based on pharmacologic characteristics and supportive initial evidence for the impact of altered kidney function on the likelihood of elevated cefepime concentrations, we next interrogated the relationship between AKI and cefepime exposure. Fifty-four patients (62%) sustained some degree of AKI during the study period, of which half (*n* = 27) were severe. Thirty-one patients (36% of the study cohort, 57% of those with AKI) had AKI present prior to starting cefepime, and for 21 patients (39% of those with AKI), this was the highest stage achieved throughout the study. Nineteen patients (35% of those with AKI) reached their peak AKI stage on study day 1, 5 (9% of those with AKI) peaked on study day 2, and only 9 patients (17% of those with AKI) had their peak AKI stage occur on day 3 or later (Supplemental Fig. 1). While both peak AKI and first detected *C*_min_ ≥ 30 tended to occur early during the study course, there was no consistent relationship between the day of peak AKI and the day of first *C*_min_ ≥ 30. Nearly half of the 13 patients who ever had elevated *C*_min_ (6 patients, 46%) had their peak AKI and first elevated *C*_min_ on the same day, while those who did not were more likely to have first detected *C*_min_ ≥ 30 prior to peak AKI stage (4 patients) rather than after peak AKI (2 patients) or in the absence of an AKI (1 patient). Patients who sustained an AKI had significantly lower cefepime clearance (*p* = 0.024) and higher total drug exposure, as measured by AUC_24_ (*p* < 0.001) and *C*_min_ (*p* < 0.001) (Table 2). In pairwise comparisons for AUC_24_ and *C*_min_, both mild (stage 1) and severe (stages 2–3) AKI differed from those without AKI (Fig. 1).

**Table 2:** Pharmacokinetic parameters of patients, by maximum AKI stage during study observation. Patient AKI stage was defined by KDIGO creatinine only criteria, and patients were categorized by the maximum AKI stage at any point during study observation. Results are reported as median (IQR) for non-normally distributed variables and *n* (%) for categorical variables. No AKI vs. stage 1 AKI vs. severe AKI were analyzed by one-way ANOVA on ranks for continuous and chi-squared analysis for categorical variables. *Vd*, volume of distribution; *CL*, clearance; *AUC*_*24*_, area under the curve over 24 h; *C*_*min*_, minimum concentration immediately prior to next cefepime dose

	Entire cohort (*n* = 87)	No AKI (*n* = 33)	Stage 1 (*n* = 27)	Severe AKI (*n* = 27)	Comparison (*p* value)
Vd, normalized (L/70 kg)	28.6 (24.9–32.9)	29.7 (24.9–35.1)	28.5 (24.4–30.9)	28.3 (25.3–33.9)	0.64
CL, normalized (L/h/70kg^0.75^)	6.4 (5.1–8.0)	7.2 (5.4–10.7)	6.0 (5.3–7.0)	6.0 (4.8–7.0)	0.024
Mean AUC_24_ (mg h/L)	929 (717–1249)	747 (634–976)	963 (857–1116)	1196 (782–1420)	< 0.001
Highest daily *C*_min_ (mg/L)	9.13 (4.6–17.9)	4.63 (2.3–12.5)	7.96 (5.7–16.3)	14.8 (9.1–23.7)	< 0.001
Any *C*_min_ > 30 mg/L, *n* (%)	13 (15%)	1 (3%)	4 (15%)	8 (30%)	0.016

To investigate whether AKI is independently associated with abnormal cefepime exposure, we created multivariable linear regression models investigating the effects of age, severity of illness (as identified by PRISM score), pre-hospitalization creatinine clearance, and severity of AKI during acute illness on AUC and *C*_min_. Age was not a significant covariate in any model; however, the PRISM score did emerge as a significant covariate for both AUC and *C*_min_ in these models. Baseline eGFR was significant for *C*_min_ but not for AUC, while the maximum AKI stage remained highly significant for both in this multivariate model. All AKI stages were significant predictors for *C*_min_, while only severe AKI, not stage 1 AKI, retained significance in predicting AUC (Table 3).

**Table 3:** Significance of covariates in cefepime mixed model analysis. The significance of each covariate is presented with its *p* value

Variable	AUC, *p* value	*C*_min_, *p* value
Age in years	0.62	0.06
PRISM	0.009	0.038
Baseline eGFR	0.07	0.03
AKI stage	0.007	0.001
No AKI vs stage 1	0.209	< 0.001
No AKI vs stage 2/3	0.002	0.002
Stage 1 vs stage 2/3	0.011	0.011

### Cefepime dosing

To assess whether the increased cefepime exposure in the severe AKI cohort reflected missed opportunities to adjust drug dosing based on eGFR per current guidelines, we reviewed the cefepime dosing regimens of these patients. Overall, 17/27 (63%) patients who had KDIGO stages 2–3 AKI received cefepime dosing appropriate for their CKiD estimated nadir eGFR, although notably only 13 of 27 (48%) had a nadir GFR ≤ 60 mL/min/1.73 m^2^ (Table S4). Among eight severe AKI patients with at least one elevated cefepime *C*_min_, one case (patient 26) occurred in a patient for whom no reduction would be recommended, one case (patient 13) occurred in a patient receiving appropriate dosing for reduced GFR, and one case (patient 20) followed intra-operative dosing. One case (patient 21) occurred when a patient was receiving a dosing higher than recommended for the nadir GFR but had no further *C*_min_ > 30 following the change to appropriate GFR-based dosing. The remaining four cases (patients 4, 16, 19, 24) occurred while receiving dosing which differed from the recommendation based on their nadir eGFR, although notably patient 4 received dosing which was appropriate based on bedside Schwartz eGFR estimate (CKiD equations had not been released at the time of this patient’s hospitalization) and patient 24 received the recommended total daily dose. This heterogeneity may suggest multiple pathways to high exposure. Some cases appear to represent missed opportunities for recognizing and adhering to eGFR-based dose adjustments, but others occur despite appropriate GFR-based dosing.

## Discussion

In this cohort of critically ill PICU patients, AKI during critical illness was associated with decreased cefepime clearance and increased metrics of cefepime exposure. Among patients with severe AKI, high cefepime concentrations sometimes occurred even with dosing which would be appropriate for their predicted GFR based on drug information references. The clinical consequences of such exposures in pediatric patients remain unknown.

Children who sustain AKI during critical illness may be vulnerable to altered drug exposure as this novel insult may have delayed recognition. Notably, AKI remains independently associated with abnormal cefepime exposure even after adjusting for pre-illness kidney function and overall severity of illness. While this is expected given the kidney elimination of cefepime, it is to our knowledge the first specific examination of risk factors for elevated cefepime exposure in pediatrics. We did not find a significant impact of BMI or duration of cefepime therapy on the risk of elevated cefepime exposure in univariate analysis. There was no impact of age in either univariate or multivariate analyses. The severity of illness was identified as a significant covariate, which could represent the impact of circulatory dysfunction, alterations in body water composition, or as-yet-unidentified features. However, AKI at the time of admission does contribute to the PRISM score, which was unable to be accounted for in our analysis and may therefore contribute to collinearity between these variables. Finally, some patients with elevated cefepime concentrations did so while receiving dosing in accordance with current dosing support recommendations. This may represent the impact on cefepime exposures of unidentified features besides GFR which differ between AKI and non-AKI groups, or may suggest that there are populations of critically ill patients in whom current methods of estimating function and relating it to drug exposure are insufficient and who would therefore benefit from precision dosing strategies.

The clinical impact, if any, of higher cefepime concentrations in critically ill pediatric patients remains unknown. It is uncertain whether adult thresholds would apply to pediatric patients, given differences in clearance, body composition, transport across the blood–brain barrier, and CNS sensitivity [34]. Critically ill patients could have additional vulnerabilities related to infection, inflammation, surgical interventions, or implanted devices, which to our knowledge have not been explicitly interrogated in adult studies. Our choice of threshold for “abnormal exposure” was based on a single adult study [22], since the adult literature lacks consensus about concentrations which confer risk of toxicity, and it represented a midpoint among thresholds suggested by different studies [21–23, 35]. Even if specific thresholds may not be directly translatable to pediatrics, we hypothesize that a dose–response relationship would continue to apply, with patients with higher concentrations having higher risks of medication-related effects compared to their peers with lower exposure. A recent adult study identified both AUC_24_ and highest daily *C*_min_ as strong predictors of neurotoxicity [23], supporting the clinical relevance of the measures of exposure chosen here. Furthermore, the risk factors identified here regarding pediatric cefepime pharmacokinetics may suggest that future neurotoxicity studies could focus on patients with kidney dysfunction as a high-risk group.

There are several limitations to this study. First and most notably, as a retrospective observational study, it is subject to the reliance on the accuracy of EMR charting for the timing of medication administration and blood draws, system-identified race, completeness of medical history, and comorbidities. Our use of medical records also precluded using oliguria criteria for AKI diagnosis, which may lead to underestimating the incidence or severity of AKI [36]. Furthermore, AKI is separate from, though related to, absolute kidney function. Given that half of the severe AKI patients in this study retained normal or minimally reduced kidney function (eGFR > 60 mL/min/1.73m^2^) which would be predicted to be sufficient drug clearance without dose adjustments, it is possible that there are additional confounders beyond nadir eGFR affecting drug exposure in AKI patients which were unable to be identified by this study. Fluid balance is one such possible confounder; while the presence and severity of fluid accumulation play an important role in drug pharmacokinetics, our ability to collect standardized measurements of fluid accumulation in this retrospective cohort was limited. All creatinine-based pediatric eGFR estimation methods carry appreciable limitations, and we have chosen to estimate eGFR using recently released Chronic Kidney Disease in Children (CKiD) estimating equations. These equations were developed for outpatients aged 1–25 years with stable chronic kidney disease, which may limit generalizability to acutely ill inpatients or patients outside this age range. However, none of the comparisons had significant changes when choosing an alternate eGFR estimating method (Supplemental Figure S2). Additionally, serum creatinine is an imperfect surrogate for glomerular filtration and will tend to estimate high glomerular filtration in patients with low muscle mass such as those with muscular dystrophy [37], mobility impairment [38], or oncologic diagnoses [39], all of which are common in the PICU. Future prospective studies may wish to use supplemental serum markers of kidney function such as cystatin C as well as urinary biomarkers such as neutrophil gelatinase-associated lipocalin (NGAL) or kidney injury molecule 1 (KIM-1). Finally, our study included 17 young adult patients over the age of 18, which, while representative of current trends in PICU admissions, further contributes to the heterogeneity of the studied population.

## Conclusions

Patients with acquired kidney dysfunction during critical illness have higher rates of elevated cefepime exposure, in some cases despite appropriate eGFR-based prescriptions. These patients represent a high-risk group to experience dose-dependent medication effects such as cefepime-induced neurotoxicity and should be subjects for future studies regarding the clinical consequences of such exposure. If excessive cefepime exposure does prove to contribute to neurotoxicity, the implementation of clinical drug monitoring protocols will require institutional adoption and validation of quantitation tests performed and resulted within clinically relevant timeframes.

## Supplementary Information

Below is the link to the electronic supplementary material.

Supplementary file1 (DOCX 214 kb)

Graphical abstract (PPTX 704 kb)

## Data Availability

De-identified data can be made available upon request by directly contacting the authors at sonya.tanggirdwood@cchmc.org.
