# Supplementary material for: Acute kidney injury is associated with abnormal cefepime exposure among critically ill children and young adults
Source: Pediatr Nephrol. 2024 Aug 16;40(2):513–21. doi: 10.1007/s00467-024-06477-4 (PMC11666613; doi:10.1007/s00467-024-06477-4)
Supplement: Supplementary file 1 [file 467_2024_6477_MOESM1_ESM.docx]

**Table of Contents:**

Page	Contents

2 	Table S1: Demographics including patients with stage 3-5 chronic kidney disease 

3	Table S2: Demographics of patients <18 years of age

4	Table S3: Institutional cefepime dosing recommendations 

5	Table S4:  Nadir renal function and cefepime dosing of patients with severe AKI

6	Figure S1: Timing of first day with elevated Cmin related to first day of peak AKI stage

7	Figure S2:  Comparison of estimates using CKID-U25 vs Bedside Schwartz equation for eGFR estimation

**Table S1: Demographics including patients with stage 3-5 chronic kidney disease.** Results are reported as median (interquartile range) for non-normally distributed continuous variables, and n (%) for categorical variables. Groups analyzed by Mann Whitney test for continuous and Chi-squared or Fischer exact analysis for categorical variables. Weight was recorded at PICU admission. BMI was calculated using CDC BMI percentile growth charts. Baseline creatinine represents lowest within 3 months prior to hospitalization. Chronic kidney disease (CKD) diagnoses categorized as recorded in the EMR at the time of ICU admission. Ventilator days represent invasive or non-invasive positive pressure ventilation. For patients with baseline positive pressure ventilation, duration of support level greater than their baseline is reported. Vasopressor days include any day on which the patient received continuous infusions of epinephrine, norepinephrine, dopamine, vasopressin, or milrinone. eGFR: estimated glomerular filtration rate. LOS: length of stay. PICU: pediatric intensive care unit. PRISM: Pediatric RISk of Mortality.

|  | **All patients** **(N=92)** | **At least 1** **Cmin  ≥30 mg/L** **(n=18)** | **All** **Cmin <30 mg/L** **(n=74)** | **Comparison (p value)** |
| --- | --- | --- | --- | --- |
| **Demographics** |  |  |  |  |
| Age, years | 8.4 (2.0-16.5) | 11.7 (3.9-19.7) | 7.5 (2.0-16.0) | 0.36 |
| Weight, kg | 26.8 (12.7-54.3) | 32.6 (15.1-47.4) | 23.8 (12.6-56.0) | 0.97 |
| BMI category |  |  |  |  |
| Underweight, n (%) | 10 (11%) | 2 (11%) | 8 (11%) | 0.89 |
| Healthy weight, n (%) | 52 (57%) | 11 (61%) | 41 (55%) |  |
| Overweight or obese, n (%) | 30 (32%) | 5 (28%) | 25 (34%) |  |
| Sex, identified at birth n, male (%) | 53 (58%) | 11 (61%) | 42 (57%) | 0.88 |
| Race/Ethnicity |  |  |  |  |
| White, n (%) | 65 (71%) | 15 (83%) | 50 (68%) | 0.19 |
| Non-white or unknown, n (%) | 27 (29%) | 3 (17%) | 24 (32%) |  |
| Baseline Creatinine (mg/dL) | 0.26 (0.17-0.46) | 0.46 (0.19-0.74) | 0.26 (0.16-0.38) | **0.009** |
| Baseline eGFR by bedside Schwartz (mL/min/1.73m^2^) | 171.3 (138.0-229.4) | 126.0 (77.6-171.6) | 188.2 (145.0-236.8) | **0.001** |
| Oncologic diagnosis | 24 (26%) | 4 (22%) | 20 (27%) | 0.77 |
| Solid organ or bone marrow transplant | 21 (23%) | 9 (50%) | 12 (16%) | **0.004** |
| Any Pre-existing CKD (stage 2-5) | 7 (8%) | 5 (28%) | 2 (3%) | **0.003** |
| **Characteristics of Hospitalization** |  |  |  |  |
| Hospital LOS, days | 28 (11.0-67.3) | 21.5 (14-85) | 30 (11-66) | 0.78 |
| PICU LOS, days | 7.5 (3.0-16.8) | 7 (2-17) | 7.5 (3-17) | 0.91 |
| Ventilator days | 2.5 (0-12) | 3.5 (0-14.5) | 2 (0-11) | 0.82 |
| Vasopressor days | 0 (0-1) | 1 0-1.25) | 0 (0-1.25) | 0.28 |
| Any AKI | 58 (63%) | 16 (89%) | 42 (57%) | **0.011** |
| Stage 2-3 AKI | 29 (32%) | 10 (56%) | 19 (26%) | **0.014** |
| PRISM score | 5 (2-11) | 8 (3.5-14.5) | 4 (2-10.2) | 0.11 |
| Total duration cefepime therapy, days | 6 (3-8) | 7 (4-11) | 5 (3-8) | 0.19 |
| 28-day mortality | 5 (5%) | 2 (11%) | 3 (4%) | 0.25 |

**Table S2: Patient demographics and features of hospitalization for patients <18 years of age.** Results are reported as median (interquartile range) for non-normally distributed continuous variables, and n (%) for categorical variables. Groups analyzed by Mann Whitney test for continuous and Chi-squared or Fischer exact analysis for categorical variables. Weight was recorded at PICU admission. BMI was calculated using CDC BMI percentile growth charts. Baseline creatinine represents lowest within 3 months prior to hospitalization. Ventilator days represent invasive or non-invasive positive pressure ventilation. For patients with baseline positive pressure ventilation, duration of support level greater than their baseline is reported. Vasopressor days include any day on which the patient received continuous infusions of epinephrine, norepinephrine, dopamine, vasopressin, or milrinone. eGFR: estimated glomerular filtration rate. LOS: length of stay. PICU: pediatric intensive care unit. PRISM: Pediatric RISk of Mortality.

|  | **All patients** **(N=70)** | **At least 1** **Cmin  ≥30 mg/L** **(n=7)** | **All** **Cmin <30 mg/L** **(n=63)** | **Comparison (p value)** |
| --- | --- | --- | --- | --- |
| **Demographics** |  |  |  |  |
| Age, years | 5.18 (1.4-12.7) | 5.7 (0.27-10.1) | 5.1 (1.46-13.3) | 0.30 |
| Weight, kg | 18.6 (11.6-47.5) | 16.5 (6.6-28.7) | 19.0 (11.9-49.8) | 0.37 |
| BMI category |  |  |  |  |
| Underweight, n (%) | 6 (9%) | 1 (14%) | 5 (8%) | 0.75 |
| Healthy weight, n (%) | 40 (58%) | 3 (42%) | 37 (59%) |  |
| Overweight or obese, n (%) | 24 (36%) | 3 (42%) | 21 (33%) |  |
| Sex, identified at birth n, male (%) | 36 (51%) | 4 (57%) | 32 (51%) | 1.0 |
| Race/Ethnicity |  |  |  |  |
| White, n (%) | 48 (70%) | 6 (86%) | 43 (68%) | 0.66 |
| Non-white, multiple, or unknown, n (%) | 23 (30%) | 1 (14%) | 20 (31%) |  |
| Baseline Creatinine (mg/dL) | 0.22 (0.15-0.35) | 0.17 (0.16-0.33) | 0.23 (0.14-0.35) | 0.45 |
| Baseline eGFR by CKiD U25 (mL/min/1.73m^2^) | 161 (133-219) | 130 (112-261) | 167 (138-215) | 0.34 |
| Oncologic diagnosis | 18 (26%) | 2 (29%) | 16 (25%) | 1.0 |
| Solid organ or bone marrow transplant | 13 (19%) | 2 (29%) | 11 (17%) | 0.60 |
| **Characteristics of Hospitalization** |  |  |  |  |
| Hospital LOS, days | 30 (11-68) | 20 (6-133) | 30 (11-68) | 0.55 |
| PICU LOS, days | 8 (4-21) | 9 (4-26) | 8 (4-21) | 0.71 |
| Ventilator days | 4 (1-15) | 4 (1-20) | 2 (0-11) | 0.35 |
| Vasopressor days | 0.5 (0-2) | 1 (1-2) | 0 (0-2) | 0.12 |
| Any AKI | 44 (63%) | 7 (100%) | 37 (58%) | **0.04** |
| Stage 2-3 AKI | 22 (31%) | 5 (71%) | 17 (27%) | **0.028** |
| PRISM score | 4 (2-11) | 16 (2-20) | 4 (2-11) | 0.10 |
| Total duration cefepime therapy, days | 5 (3-8) | 7 (3-11) | 5 (3-8) | 0.62 |
| 28-day mortality | 4 (6%) | 2 (29%) | 2 (3%) | **0.047** |

**Table S3: Institutional guidelines for cefepime dosing.**

*Pseudomonas* spp. infections (suspected or proven): IM, IV: 50 mg/kg/dose every 8 hours; maximum dose: 2,000 mg/dose 

*Febrile neutropenia, empiric therapy:* Infants, Children, and Adolescents: IV: 50 mg/kg/dose every 8 hours; maximum dose: 2,000 mg/dose duration of therapy dependent upon febrile neutropenia risk-status;

Cefepime dosing adjustments for maintenance dose in renal impairment:

|  | Usual dose: q8 schedule |
| --- | --- |
| >60 mL/minute/1.73 m^2^ | 50 mg/kg/dose every 8 hours; maximum dose: 2,000 mg/dose |
| 30 to 60 mL/minute/1.73 m^2^ | 50 mg/kg/dose every 12 hours; maximum dose: 2,000 mg/dose |
| 11 to 29 mL/minute/1.73 m^2^ | 50 mg/kg/dose every 24 hours; maximum dose: 2,000 mg/dose |
| <11 mL/minute/1.73 m^2^ | 25 to 50 mg/kg/dose every 24 hours; maximum dose: 1,000 mg/dose |

Source: Cefepime [CCHMC formulary of medications]. *Lexi-Drugs*. UpToDate Lexidrug. UpToDate Inc.

**Table S4:** **Renal function and cefepime dosing of patients with severe AKI.** Patients are numbered in order of study enrollment. Nadir eGFR is calculated using CKiD U25 equations and the highest creatinine recorded during the study. eGFR nadir are shaded according to dose reduction recommendations described in Table S3 (white  >60 mL/minute/1.73 m^2^, light grey 30-60, dark grey 11-29). Cefepime dosing schedule is shown at the time of abnormal Cmin (when relevant) or study enrollment (when all Cmin <30). Bold outlines around a dosing schedule indicate one which differs from the institutional recommendations for their nadir eGFR. Dosing adjustments during study period are explained in the notes. 

| **Patient #** | **eGFR nadir** **(mL/min/1.73m**^**2**^**)** | **Instances of C**_**min**_ **≥30** | **Cefepime dosing** | **Dosing appropriate for nadir GFR?** | **Notes** |
| --- | --- | --- | --- | --- | --- |
| 1 | 83 |  | 50/kg q8 | Y |  |
| 2 | 48 |  | 2g q8 | N |  |
| 3 | >90 |  | 50/kg q8 | Y |  |
| 4 | 27 | 2 | 50/kg q12 | N |  |
| 5 | 84 |  | 2g q8 | Y |  |
| 6 | 87 |  | 50/kg q8 | Y |  |
| 7 | 75 |  | 50/kg q8 | Y |  |
| 8 | 52 |  | 2g q12 | Y |  |
| 9 | >90 |  | 50/kg q8 | Y |  |
| 10 | 60 |  | 50/kg q8 | N |  |
| 11 | >90 |  | 2g q8 | Y |  |
| 12 | >90 |  | 50/kg q8 | Y |  |
| 13 | 52 | 2 | 2g q12 | Y |  |
| 14 | 43 |  | 2g q8 | N |  |
| 15 | >90 |  | 2g q8 | Y |  |
| 16 | 60 | 3 | 2g q8 | N |  |
| 17 | >90 |  | 50/kg q8 | Y |  |
| 18 | 37 |  | 50/kg q12 | Y |  |
| 19 | 10 | 5 | 50/kg q12 | N | Started 50/kg q8, reduced to 50/kg q12 following AKI. All abnormal cefepime levels occurred while receiving 50/kg q12 |
| 20 | >90 | 1 | 50/kg q8 | other | Single elevated Cmin followed intra-operative dosing.  No further Cmin ≥30 |
| 21 | 44 | 1 | 2g q8 | N | Dose adjusted to 2g q12 upon recognition of AKI. No further Cmin ≥30 following adjustment |
| 22 | 70 |  | 2g q8 | Y |  |
| 23 | 71 |  | 50/kg q8 | Y |  |
| 24 | 19 | 6 | 1g q12 | N | equivalent total daily dose to recommended 2g q24h |
| 25 | 55 |  | 50/kg q8 | N |  |
| 26 | 77 | 2 | 50/kg q8 | Y |  |
| 27 | 54 |  | 50/kg q8 | N |  |

**Figure S1:** **Timing of first day with elevated Cmin related to first day of peak AKI stage.** Study day 1 represents the date of first antibiotic administration in the ICU. AKI stage was determined by comparing daily creatinine with patient baseline according to KDIGO creatinine criteria. Percentages represent fraction of total group (n=54 patients with AKI, n-13 patients with Cmin ≥30). 

 

**Figure S2: Comparison of the effects of using Schwartz vs CKiD U25 methods for estimating pediatric eGFR from serum creatinine on overall demographics (A), mixed model (B), and dosing analysis of AKI patients (C).** For both eGFR methods, pre-illness eGFR does not significantly differ between groups who ever have Cmin ≥30 as compared to those who do not and the variables which emerged as significant in the mixed models for AUC and Cmin were the same. While the absolute differences in nadir eGFR are small, in 2 cases the choice of method changes the dosing category, from “moderate” to “severe” reduction in function in patient 4 and from “no reduction” to “mild” reduction in function in patient 10. Notably in both cases the absolute difference between estimates is <10 mL/min/1.73 m2, and the patient received dosing appropriate for their Schwartz-derived eGFR.
